# Risk factors and treatment outcomes of 239 patients with testicular granulosa cell tumors: a systematic review of published case series data

**DOI:** 10.1007/s00432-020-03326-3

**Published:** 2020-07-27

**Authors:** Josias Bastian Grogg, Kym Schneider, Peter-Karl Bode, Benedikt Kranzbühler, Daniel Eberli, Tullio Sulser, Joerg Beyer, Anja Lorch, Thomas Hermanns, Christian Daniel Fankhauser

**Affiliations:** 1 Department of Urology, University Hospital, University of Zurich, Frauenklinikstrasse 10, 8091 Zurich, Switzerland; 2 Department of Pathology of Molecular Pathology, University Hospital, University of Zurich, Zurich, Switzerland; 3 Department of Oncology, University Hospital, University of Bern, Bern, Switzerland; 4 Department of Oncology, University Hospital, University of Zurich, Zurich, Switzerland

**Keywords:** Interstitial cell tumors, Testis cancer, Granulosa

## Abstract

**Purpose:**

Testicular granulosa cell tumors (tGrCT) are rare sex cord-stromal tumors. This review aims to synthesize the available evidence regarding the clinical presentation and clinicopathological characteristics, treatment and outcomes.

**Methods:**

We conducted a systematic literature search using the most important research databases. Whenever feasible, we extracted the data on individual patient level.

**Results:**

From 7863 identified records, we included 88 publications describing 239 patients with tGrCT. The majority of the cases were diagnosed with juvenile tGrCT (166/239, 69%), while 73/239 (31%) patients were diagnosed with adult tGrCT. Mean age at diagnosis was 1.5 years (± 5 SD) for juvenile tGrCT, and 42 years (± 19 SD) for adult tGrCT. Information on primary treatment was available in 231/239 (97%), of which 202/231 (87%) were treated with a radical orchiectomy and 20/231 (9%) received testis sparing surgery (TSS). Local recurrence after TSS was observed in 1/20 (5%) cases. Metastatic disease was never observed in men with juvenile tGrCT but in 7/73 (10%) men with adult tGrCT. In 5/7 men with metastatic tGrCT, metastases were diagnosed at initial staging, while 2/7 patients developed metastases after 72 and 121 months of follow-up, respectively. Primary site of metastasis is represented by the retroperitoneal lymph nodes, but other sites including lungs, liver, bone and inguinal lymph nodes can also be affected. In comparison with non-metastatic adult tGrCT, men with metastatic adult tGrCT had significantly larger primary tumors (70 vs 24 mm, *p* 0.001), and were more likely to present with angiolymphatic invasion (57% vs 4%, *p* 0.002) or gynecomastia (29% vs 3%, *p* 0.019). In five out of seven men with metastatic disease, resection of metastases or platinum-based chemotherapy led to complete remission.

**Conclusion:**

Juvenile tGrCT represent a benign entity whereas adult tGCTs have metastatic potential. Tumor size, presence of angiolymphatic invasion or gynecomastia represent risk factors for metastatic disease. The published literature supports the use of testis sparing surgery but there is only limited experience with adjuvant therapies. In the metastatic setting, the reviewed literature suggests that aggressive surgical and systemic treatment might cure patients.

**Electronic supplementary material:**

The online version of this article (10.1007/s00432-020-03326-3) contains supplementary material, which is available to authorized users.

## Introduction

Testicular granulosa cell tumors (tGrCT) are a rare group of sex cord-stromal tumors (SCST) originating from epithelial elements of the sex cord. While they represent the most common SCST in the ovary (Young 2005), the testicular manifestation was only reported sporadically since the first description in 1952 (Laskowski 1952). According to the current World Health Organization (WHO) classification of Tumors of the Urinary System and Male Genital Organs, two histologic subtypes are distinguished: The juvenile tGrCT and the less frequent adult tGrCT (Idrees et al. 2017). While the juvenile subtype accounts for 6% of all prepubertal testicular tumors and represents the most frequent congenital testicular tumor (Kao et al. 2015), the adult tGrCT is rare and only reported in small case series and case reports. For both histological subtypes, the risk of metastatic spread is ill defined (Cecchetto et al. 2010; Mostofi et al. 1959).

Due to the rarity of tGrCT, there are several unanswered questions regarding the optimal management of patients with localized or metastatic tGrCT. The aim of this systematic literature review was to provide an overview of the available data on tGrCT patients, regarding clinical presentation, clinicopathologic factors predicting metastatic disease, experience with testis sparing surgery, sites of metastasis, and outcome and treatment success in case of metastatic disease.

## Methods

### Evidence acquisition

#### Data acquisition and search strategy

This systematic literature review was conducted based on the Preferred Reporting Items for Systematic Reviews and Meta-analysis (PRISMA) statement (Moher et al. 2009, 2015). Prior to data acquisition, the review protocol and search strategy were published on the University of York’s PROSPERO registry (http://www.crd.york.ac.uk/PROSPERO; registration number CRD42018110112).

Our literature search identified articles published up to 5th May 2018 and covered the most significant electronic databases (MEDLINE, EMBASE, Scopus, Cochrane Database of Systematic Reviews and Web of Science). A clinical medical librarian applied a broad approach using several combinations, synonyms and search terms related to “granulosa cell tumor”, “sex cord tumor”, “stromal tumor” or “interstitial cell tumor” to identify all relevant articles. Non-English literature was excluded unless the abstract was available in English or the full text in French, Spanish, Italian or German. The reference lists of the identified publications were screened manually to identify additional studies. A detailed description of our search strategy is shown in Appendix 1.

Deduplication of the resulting list of publications was achieved automatically using the close match function of our reference management software. The remaining duplicate articles were identified via manual deduplication done by two authors (JG, KS). The same authors screened the titles and abstracts independently to select publications that fulfilled the eligibility criteria and came to a consensus about the inclusion of those studies. Data of the same study that appeared in multiple publications were counted only once in the synthesis. Disagreements were discussed and resolved by consensus or by third-party arbitration (CDF). Any case reports, clinical case series and other reports describing patients with juvenile or adult tGrCT were included.

#### Types of outcome measures included

Studies reporting clinical or pathological variables, treatment of local or metastatic disease, site of metastases, disease-free, cancer-specific or overall survival were eligible for this review. To capture all relevant literature, our search strategy did not include predefined interventions, controls or outcomes.

#### Data extraction

Based on the Cochrane Consumers and Communication Review Group’s data extraction template, a data extraction sheet was developed and improved after a pilot-testing phase on twenty randomly selected eligible studies. Data on study design, patient characteristics, clinicopathological risk factors, treatment and follow-up were collected. Whenever feasible, data were gathered on single patient level.

#### Statistical analysis

Receiver operating curve (ROC) analyses using the maximal Youden’s index (= Sensitivity + Specificity − 1) (Youden 1950) were used to determine an optimal cut-off value of continuous variables. 2 × 2 table analysis was performed with R version 3.3.1 (R Foundation for Statistical Computing, Vienna, Austria) using the package *epiR*. Descriptive data are presented as median and interquartile range (IQR). Weighted medians were used to estimate already statistically processed data of cohort studies as well as individual patient data of single case reports. The results for continuous normally distributed variables are expressed as mean ± standard deviation (SD). Continuous non-normally distributed variables are presented as median and interquartile ranges (IQR) and categorical variables are presented as percentage. Categorical data were compared using the Chi-square test of independence. Normally distributed continuous variables were analyzed using the independent samples *t* test, while the Mann–Whitney *U* test was used for non-normally distributed continuous variables. All *p* values < 0.05 were considered statistically significant. All statistical tests were two sided.

### Evidence synthesis

#### Studies

**Fig. 1:**
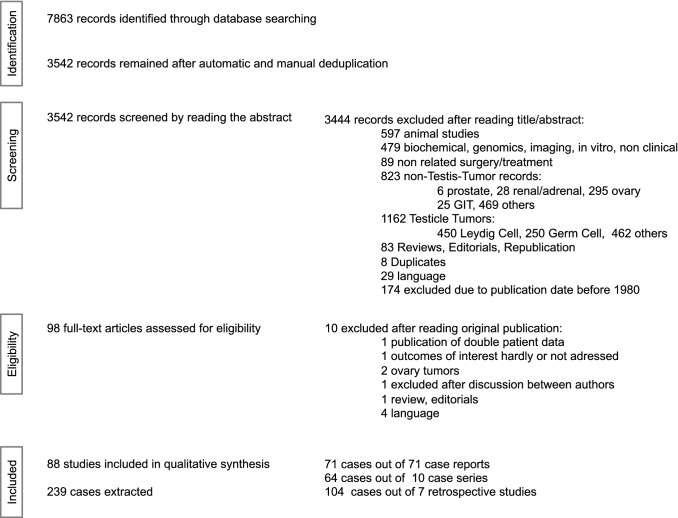
Flowchart of the study selection process

After automated and manual deduplication, 98 of 3542 publications which met the initial search criteria were eligible for full-text review after screening of title and abstract. We finally included 88 studies, resulting in a dataset of 239 patients (Fig. 1). Data regarding follow-up were available in 185 of 239 (77%) patients. Mean follow-up time was 48 months (± 40 SD).

#### Subgroups, demographics, clinical symptoms and laboratory findings

We identified 239 cases of tGrCT, of which 166 (69%) presented with the histological variant juvenile tGrCT and 73 (31%) with adult tGrCT (Table 1). Juvenile tGrCT were diagnosed at a mean age of 1.5 years (± 5 SD), with single reports of diagnosis in early adulthood (27 and 34 years) (Gravas et al. 2007; Lin et al. 2008). Patients with adult tGrCT were diagnosed at a mean age of 42 years (± 19 SD). Overall, data about clinical presentation were available in 171 of the 239 patients (72%). The majority of patients presented with a testicular mass (78/171, 46%) or testicular enlargement (71/171, 42%), whereas scrotal pain was only described in 8/171 (5%) cases. Alpha-fetoprotein (AFP) was elevated in 18/171 (11%) of the cases, whereas other tumor markers like human chorionic gonadotropin (HCG) and lactate dehydrogenase (LDH) were elevated in only 3/171 (2%) and 2/171 (1%) patients, respectively. Hormonal changes were reported in 19/171 (11%), including gynecomastia (7/171, 4%) and anomaly of puberty (2/171, 1%). History of ipsi- or contralateral cryptorchidism was reported in 18/166 (11%) of juvenile tGrCT and in 3/73 (4%) of adult tGrCT.

**Table 1:** Patient characteristics

	Individual patient level of 81 reports*			Study-level data from 88 reports
	All testicular granulosa cell tumors (%)	Juvenile granulosa cell tumors	Adult granulosa cell tumors	All testicular granulosa cell tumors
Number of patients	138	65	73	239
Age (years)				
Mean (± SD)	23 (± 25)	1.5 (± 5)	42.0 (± 19)	23 (± 25)
Available	137 (99)	65 (100)	72 (99)	
Missing	1 (1)	0 (0)	1 (1)	
Size (mm)				
Median (IQR)	23 (14–44)	20 (13–38)	26 (15–45)	21 (15–44)
Available	100 (72)	36 (55)	64 (88)	
Missing	38 (28)	29 (45)	9 (12)	
Side				
Available	133 (96)	61 (94)	72 (99)	
Missing	5 (4)	4 (6)	1 (1)	
Left	62 (46)	24 (39)	38 (53)	101 (42)
Right	66 (50)	33 (54)	33 (46)	92 (38)
Bilateral	5 (4)	4 (7)	1 (1)	5 (2)
Clinical presentation				
Available	104 (75)	63 (97)	41 (56)	171 (72)
Missing	34 (25)	2 (3)	32 (44)	68 (28)
Testicular enlargement	45 (43)	24 (38)	21 (51)	71 (42)
Testicular mass	37 (36)	22 (35)	15 (37)	78 (46)
Hormonal changes	13 (13)	9 (14)	4 (10)	19 (11)
Abdominal mass	7 (7)	7 (11)	–	9 (5)
Gynecomastia	6 (6)	2 (3)	4 (10)	7 (4)
Incidental finding	9 (9)	6 (10)	3 (7)	9 (5)
Scrotal pain	8 (8)	2 (3)	6 (15)	8 (5)
Anomaly of puberty	2 (2)	2 (3)	–	2 (1)
AFP elevation	12 (12)	11 (17)	1 (2)	18 (11)
bHCG	3 (3)	2 (3)	1 (2)	3 (2)
LDH	2 (2)	1 (2)	1 (2)	2 (1)
Cryptorchidism ipsilateral	10 (10)	9 (14)	1 (2)	16 (9)
Cryptorchidism contralateral	5 (5)	3 (5)	2 (5)	5 (3)

*AFP* alpha-fetoprotein, *bHCG* beta-human chorionic gonadotropin, *IQR* interquartile range, *LDH* lactate dehydrogenase, *SD* standard deviation, *IQR* inter quartile range, *this column includes only patients of which individual patient level data were available

#### Local treatment and pathological findings

In 231 of 239 cases, information about treatment was available (Table 2). Most patients underwent orchiectomy (202/231, 87%), while testis sparing surgery (TSS) was performed in 20 cases (9%). Local recurrence 3 months after TSS was reported in one patient (1/20, 5%). After receiving a salvage hemiscrotectomy, the patient remained disease free for at least 18 months (Gravas et al. 2007). Seven patients (3%) with intra-abdominal cryptorchidism underwent laparotomy. Two patients (1%) underwent testicular biopsy only without any further treatment. The median tumor size of tGCTs was 21 mm (IQR 15–44). The most frequently described pathological findings included high mitotic rate (53/239, 22%), necrosis (12/239, 5%), angiolymphatic invasion (10/239, 4%) and pleomorphism (10/239, 4%). Other features like infiltrating margins, extracapsular invasion or calcification were seen in four patients or less. The most frequently reported immunohistochemistry included vimentin (58/239, 24%), inhibin (55/239, 23%) and calretinin (24/239, 10%).

**Table 2:** Treatment and outcome of patients with juvenile and adult tGrCT

Author	Year	No. of cases	Primary treatment (no. of patients)	Location of metastatic spread (no. of patients)	Treatment of metastatic disease (no. of patients)	Histology	Mean follow-up time in months	Outcome during follow-up (no. of patients)
Summary statistics								
All tGrCT		239	140 orchiectomy, 62 gonadectomy, 20 TSS, 7 laparotomy, 2 biopsy only	5 RPLN, 1 ILN, 1 lung, 1 liver, 1 bone	3 RPLND, 4 Chemo, 1 ILND 1 RT, 1 metastasectomy		48	121 NED, 2 local recurrence, 7 died, 18 LTFU
All juvenile tGrCT		166	72 orchiectomy, 62 gonadectomy, 15 TSS, 7 laparotomy, 2 biopsy only	0	–	jGCT	54	80 NED, 1 local recurrence, 3 died
All adult tGrCT		73	68 orchiectomy, 5 TSS	5 RPLN, 1 ILN, 1 lung, 1 liver, 1 bone	3 RPLND, 4 Chemo, 1 ILND 1 RT, 1 metastasectomy	aGCT	34	41 NED, 1 local recurrence, 4 died
Single reports								
Trenti, E., et al.	2018	1	TSS			aGCT	12	NED
Tartar, T., et al.	2017	1	Orchiectomy			jGCT		
Elbachiri, M., et al.	2017	1	Orchiectomy	RPLN	Chemo (BEP)	aGCT	6	Contralateral recurrence (6)
Dundas, M., et al.	2017	1	Orchiectomy			jGCT	7	NED
Mohapatra, A., et al.	2016	1	Orchiectomy	RPLN	RPLND	aGCT	32	NED
Liu, S. and P. Koscheski	2016	1	TSS			jGCT		
Karachaliou, F., et al.	2016	1				jGCT	18	NED
Bani, M. A., et al.	2016	1	Orchiectomy			aGCT	4	NED
Al-Alao, O., et al.	2016	1	Orchiectomy			aGCT	12	NED
Vallonthaiel, A. G., et al.	2015	1	Orchiectomy			aGCT		LTFU
Kao, Chia-Sui	2015	70	62 gonadectomy, 6 TSS, 2 biopsy only			jGCT	61	24 NED
González, B. I., et al.	2015	1	Orchiectomy			jGCT		
Giulianelli, R., et al.	2015	1	Orchiectomy			aGCT	12	NED
Bani, M. A., et al.	2015	1	Orchiectomy			aGCT		LTFU
Tsitouridis, I., et al.	2014	1	Orchiectomy			aGCT	12	NED
Tanner, S. B., et al.	2014	1	Orchiectomy			aGCT		NED
Schubert, T. E. O., et al.	2014	1	Orchiectomy			aGCT		LTFU
Illescas, T., et al.	2014	1	Orchiectomy			jGCT		LTFU
Cosentino, M., et al.	2014	1	Orchiectomy			jGCT		LTFU
Cornejo, K. M. and R. H. Young	2014	32	30 orchiectomy, 2 TSS			aGCT	37	17 NED, 1 orchiectomy
Claros, O. R., et al.	2014	1	Orchiectomy			jGCT	48	NED
Aranha, A., et al.	2014	1	Orchiectomy			jGCT	24	NED
Norman, R. W., et al.	2013	1	Orchiectomy			aGCT	18	NED
Miliaras, D., et al.	2013	1	Orchiectomy			aGCT	24	NED
Partalis, N., et al.	2012	1				jGCT	12	NED
Muhlschlegel, J. M., et al.	2012	1	TSS			jGCT	24	NED
Couture, J. and S. Bolduc	2012	1	Orchiectomy			jGCT		
Bulotta, A. L., et al.	2012	1	Orchiectomy			jGCT	1	NED
Yu, D. C., et al.	2011	1	Orchiectomy			jGCT	17	NED
Tiscione, D., et al.	2011	1	TSS			aGCT		LTFU
Song, Z., et al.	2011	1	Orchiectomy			aGCT		LTFU
Zugor, V., et al.	2010	2	2 orchiectomy			jGCT	17	2 NED
Seixas-Mikelus, S. A., et al.	2010	1	Orchiectomy			jGCT	12	NED
Oscar Tapia, E., et al.	2010	1	Orchiectomy			jGCT	8	NED
Gun, F., et al.	2010	3	2 orchiectomy, 1 TSS			jGCT		
Cecchetto, G., et al.	2010	5	3 TSS			jGCT		
Peterson, C. and S. Skoog	2008	1	Orchiectomy			jGCT		LTFU
Mitra, A., et al.	2008	1	Orchiectomy			aGCT		LTFU
Lin, K.-H., et al.	2008	1	Orchiectomy			jGCT	96	NED
Kucukodaci, Z., et al.	2008	1	Orchiectomy			aGCT	10	NED
Kim, D. J., et al.	2008	1	Orchiectomy			aGCT	12	NED
Hammerich, K. H., et al.	2008	1	Orchiectomy	Lung	Chemo (BEP)	aGCT	13	NED
Gupta, A., et al.	2008	1	Orchiectomy			aGCT	12	NED
Dudani, R., et al.	2008	1	Orchiectomy			jGCT	6	NED
Yikilmaz, A. and E. Y. Lee	2007	1				jGCT		
Trobs, R. B., et al.	2007	1	Orchiectomy			jGCT	47	NED
Lopez, J. I.	2007	1	Orchiectomy			aGCT	12	NED
Gravas, S., et al.	2007	1	TSS			jGCT	18	NED, ipsilateral recurrence (3 mo)
Ditonno, P., et al.	2007	1	Orchiectomy			aGCT		LTFU
Barroca, H., et al.	2007	1	Orchiectomy			jGCT		
Alexiev, B. A., et al.	2007	1	Orchiectomy			jGCT		
Zugor, V., et al.	2006	1	Orchiectomy			jGCT	12	NED
Hisano, M., et al.	2006	1	Orchiectomy			aGCT	48	NED
Arzola, J., et al.	2006	1	Orchiectomy			aGCT	9	NED
Suppiah, A., et al.	2005	1	Orchiectomy	Bone	Metastasectomy	aGCT	4	NED
Shukla, A. R., et al.	2004	3	2 orchiectomy, 1 TSS			jGCT	78	3 NED
Guzzo, T., et al.	2004	1	Orchiectomy			aGCT		LTFU
Moore, W., et al.	2003	1	Orchiectomy			jGCT		
Fidda, N. and D. A. Weeks	2003	1	Orchiectomy			jGCT	18	NED
Fagin, R., et al.	2003	1	Orchiectomy			jGCT	6	NED
Bryan, D. E., et al.	2003	3	3 orchiectomy			jGCT	8	2 NED
Wang, B. Y., et al.	2002	1	TSS			aGCT		LTFU
Nieto, N., et al.	2002	1	Orchiectomy			jGCT	30	NED
Antunes, L., et al.	2002	1	Orchiectomy			jGCT		
Ji, E. K. and K. S. Cho	2001	1	Orchiectomy			aGCT		LTFU
Al-Bozom, I. A., et al.	2000	1	Orchiectomy			aGCT	7	NED
Harms, D. and L. R. Kock	1997	11	8 orchiectomy, 1 TSS			jGCT		8 NED, 3 LTFU
Chan, Y. F., et al.	1997	2	2 orchiectomy			jGCT	9	2 NED
Perez-Atayde, A. R., et al.	1996	6	6 orchiectomy			jGCT		6 NED, 1 died
Goswitz, J. J., et al.	1996	4	4 orchiectomy			jGCT	96	3 NED, 1 LTFU, 1 died
Berensztein, E., et al.	1995	1	Orchiectomy			jGCT		
Tanaka, Y., et al.	1994	1	Laparotomy			jGCT	72	NED
Jimenez-Quintero, L. P., et al.	1993	7	7 orchiectomy	P1: RPLN, liver P2: RPLN, ILN	P1: 1 RPLND, Chemo (CD) P2: RPLND, Chemo (E), INLD, RT	aGCT	23	3 NED, 2 died (32 mo, 134 mo)
Nistal, M., et al.	1992	1	Orchiectomy			aGCT	24	NED
May, D., et al.	1992	1	Laparotomy			jGCT	6	NED
Matoska, J., et al.	1992	1	Orchiectomy	RPLN	RPLND, RT	aGCT	168	NED
Yokoyama, J., et al.	1990	1	Orchiectomy			jGCT	24	NED
Due, W., et al.	1990	1	Orchiectomy			aGCT	2.25	Died
Chan, J. K., et al.	1990	1	Laparotomy			jGCT	30	NED
Gaylis, F. D., et al.	1989	1	Orchiectomy			aGCT		LTFU
Nistal, M., et al.	1988	1	Orchiectomy			jGCT	24	NED
Uehling, D. T., et al.	1987	1	Orchiectomy			jGCT	36	NED
Raju, U., et al.	1986	1	Laparotomy			jGCT	60	NED
Young, R. H., et al.	1985	3	3 laparotomy			jGCT	6	NED
Talerman, A.	1985	1	Orchiectomy			aGCT	36	NED
Pinto, M. M.	1985	1	Orchiectomy			jGCT		
Lawrence, W. D., et al.	1985	14	14 orchiectomy			jGCT		4 NED
Crump, W. D.	1983	1				jGCT	0	Died after birth

*aGCP* adult granulosa cell tumor, *jGCP* juvenile granulosa cell tumor, *MFS* metastatic-free survival, *RPLND* retroperitoneal lymph node dissection, *ILN* inguinal lymph nodes, *ILND* inguinal lymph node dissection, *RT* radiotherapy, *DOD* died of disease, *NED* no evidence of disease, *SD* stable disease, Chemo: *BEP* bleomycin, etoposide, cisplatin, *DC* doxorubicin–cisplatin, *E* etoposide

### Metastatic disease

While all of the 166 reported cases of juvenile tGrCT were exclusively benign, 7 out of 73 (10%) patients with adult tGrCT showed metastatic disease (Table 3). Mean age at diagnosis of patients with metastatic tGrCT was 45 years (± 14 SD). The median primary tumor size of metastatic cases was 70 mm (IQR: 51–90). Painless testicular enlargement (*n* = 3) or palpable mass (*n* = 2) was the most frequent clinical presentation in metastatic tGCTs, two patients also presented with gynecomastia. One patient was diagnosed incidentally during an ultrasound examination. None of the reported metastatic cases showed elevated AFP, HCG or LDH.

**Table 3:** Clinical and pathological features of metastatic and non-metastatic adult granulosa cell tumors

	Metastatic adult tGrCT, *n* = 7 (%)	Non-metastatic adult tGrCT, *n* = 66 (%)	*P* value
Age (years)	Available 7/7	Available 66/66	
Mean (± SD)	45 (± 14)	42 (± 19)	0.659
< 50 years	3 (43)	35 (53)	
≥ 50 years	4 (57)	31 (47)	0.223
Tumor size (mm)	Available 6/7	Available 59/66	
Median (IQR)	70 (51–90)	24 (14–42)	*0.001*
< 46 mm	0 (0)	49 (83)	
≥ 46 mm	6 (100)	10 (17)	*<* *0.001*
Tumor markers	Available 7/7	Available 35/66	
APF	0 (0)	1 (2)	
HCG	0 (0)	1 (2)	
LDH	0 (0)	1 (2)	
Clinical presentation	Available 7/7	Available 35/66	
Median time to diagnosis	7 months	24 months	0.364
Testicular enlargement	3 (43)	18 (27)	0.754
Palpable mass	2 (29)	13 (20)	0.960
Gynecomastia	2 (29)	2 (3)	*0.019*
Incidental finding	1 (14)	2 (3)	0.453
Hormonal changes	1 (14) (T↓, FSH/LH↑)	2 (3)	0.102
Infertility	0 (0)	0 (0)	–
Scrotal pain	0 (0)	6 (9)	0.316
Pathology features	Available: 7/7	Available: 47/66	
Angiolymphatic invasion	4 (57)	2 (4)	*0.002*
Necrosis	2 (29)	4 (9)	0.115
Pleomorphism	2 (29)	5 (11)	0.103
High mitotic index	2 (29)	8 (17)	0.109
Extracapsular growth	2 (29)	2 (4)	0.771
Infiltrating margins	1 (14)	0 (0)	0.299
Atypias	0 (0)	1 (2)	0.3
Staining			
Inhibin	4 (57)	13 (28)	0.641
Vimentin	3 (43)	23 (49)	0.148
Calretinin	0 (0)	8 (17)	–

Normally distributed continuous variables were analyzed using the independent samples *t* test. The Mann–Whitney *U* test was used for non-normally distributed continuous variables. The Chi-square test was used for categorical variables

*SD* standard deviation, *tGrCT* testicular granulosa cell tumor, *IQR* inter quartile range, *AFP* alpha fetoprotein, *HCG* human chorionic gonadotropin, *LDH* lactate dehydrogenase

#### Risk factors for metastatic disease

Predictive variables for metastatic disease included tumor size, angiolymphatic invasion and presence of gynecomastia (supplementary Table 1). According to ROC analyses, the ideal cut-off for tumor size to predict metastatic disease was 46 mm (AUC 0.86 95% CI 0.76–0.93). A tumor size above 46 mm was observed in 7/7 (100%) metastatic and in (10/61, 16%, *p* value: < 0.001) non-metastatic patients. Angiolymphatic invasion was more common in metastatic compared to non-metastatic disease (4/7 (57%) vs. 8/54 (15%), *p* = 0.002). Furthermore, gynecomastia was more common in metastatic compared to non-metastatic disease (29% vs. 3%, *p* = 0.019).

#### Onset of metastatic disease

**Fig. 2:**
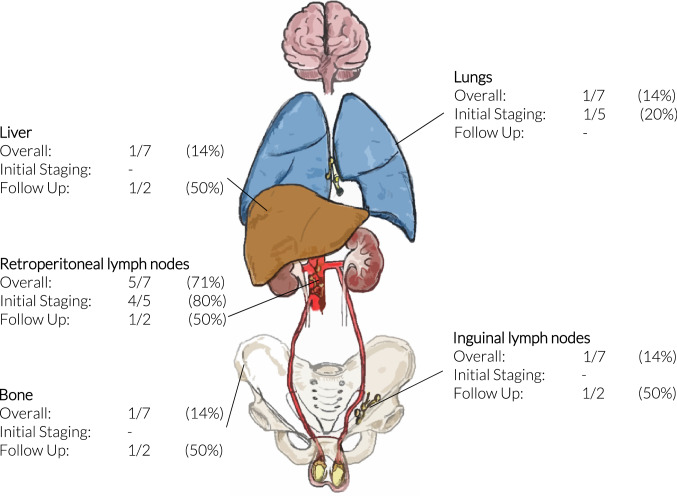
Anatomical locations of metastatic sites both at initial staging during follow-up

In five patients, metastatic disease was diagnosed at initial staging. The primary site of metastatic disease at staging included retroperitoneal lymph nodes (RPLN) in four and the chest in one patient (Fig. 2). Overall, three patients developed metastatic disease recurrence: Two patients with localized disease at staging showed metastatic recurrence (2/66, 3%) after a 72 and 121 months, respectively. Sites of recurrence included distal tibia in one case and the retroperitoneum and liver in the second case. One patient with metastatic disease in the retroperitoneum at initial staging showed recurrence in the inguinal lymph nodes later on (Table 4).

**Table 4:** Characteristics, treatment and outcomes of patients with metastatic testicular adult granulosa cell tumors

No.	Author (year)	Age, Side	Size (mm)	Clinical presentation	Local treatment	Initial site and onset of metastatic disease (months after first diagnosis)	First-line treatment of metastatic disease	Response to first-line treatment	Risk factors for metastatic disease	Follow-up (months after diagnosis)
1	Suppiah, A. (2005)	51, left	Missing	Testicular enlargement	Orchiectomy	Bone, 72	Resection	CR	None	NED (76)
2	Mohapatra, A. (2016)	57, left	47	Palpable mass, gynecomastia	Orchiectomy	RPLN, 0	RPLND	CR	Size + , gynecomastia + , angiolymphatic invasion+	NED (32)
3	Matoska, J. (1992)	26, left	100	Testicular enlargement, gynecomastia	Orchiectomy	RPLN, 0	RPLND, RT	CR	Size + , gynecomastia +, angiolymphatic invasion+	NED (168)
4	Elbachiri, M. (2017)	40, left	55	Palpable mass	Orchiectomy	RPLN, 0	Chemo (BEP)	CR	Size+	NED (6)
5	Hammerich, K. H. (2008)	55, right	105	Testicular enlargement	Orchiectomy	Lung, 0	Chemo (BEP)	CR	Size + , angiolymphatic invasion+	NED (13)
6	Jimenez-Quintero, L. P. (1993)	60, left	70	Testicular enlargement	Orchiectomy	RPLN, liver, 121	Chemo (DC)	PD	Size+	DOD (134)
7	Jimenez-Quintero, L. P. (1993)	29, right	75	Incidental	Orchiectomy	RPLN, 0, ILN 12	RPLND, Chemo (E)	PD: ILN met. (12 Mo) 2nd line: INLD, RT	Size + , angiolymphatic invasion+	SD (14)

*RPLND* retroperitoneal lymph node dissection, *ILN* inguinal lymph nodes, *ILND* inguinal lymph node dissection, *RT* radiotherapy, *DOD* died of disease, *CR* complete remission, *PD* progressive disease, *NED* no evidence of disease, *SD* stable disease, *Chemo* BEP: bleomycin, etoposide, cisplatin, *DC* doxorubicin–cisplatin, *E* etoposide

#### Treatment of metastatic disease

The median follow-up time for men with metastatic disease was 14 months (IQR: 9–83). Of seven patients with metastasized tGrCT, five patients showed complete remission after treatment (Table 4). Patient #1 had a metastatic recurrence in the distal left tibia 6 years after diagnosis and received a below knee amputation and was free of disease for at least another 4 months (Suppiah et al. 2005). Patient #2 and #3 showed metastatic disease in the RPLN at initial staging were treated with RPLND and remained without evidence of disease during a total follow-up time of 32 (Mohapatra et al. 2016) and 168 months (Matoska et al. 1992), respectively. Patient #4 with metastatic disease in the RPLN received four cycles of BEP and remained disease free for at least 6 months (Elbachiri et al. 2017). Patient #5 presented with lung metastases at initial staging, received 6 cycles of BEP and showed no evidence of disease for at least 13 months (Hammerich et al. 2008). Patient #6 was diagnosed with RPLN and liver metastases 121 months after orchiectomy. He subsequently received chemotherapy with doxorubicin–cisplatin (DC) but died of progressive disease 134 months after initial diagnosis (Matoska et al. 1992). Patient #7 underwent a modified retroperitoneal lymph node dissection, which revealed metastatic involvement of four lymph nodes. Consequently, the patient was treated with one cycle of chemotherapy with etoposide, which was discontinued because of side effects. After 2 months, ipsilateral inguinal lymph node metastases were detected. After resection and additive radiotherapy, he remained disease free for at least 14 months (Matoska et al. 1992).

## Discussion

Our systematic review of published case series represents the most comprehensive summary of the available literature regarding tGCTs providing recommendation for (1) local therapy, (2) risk factors for metastatic disease and recommendations for follow-up and (3) treatment of metastatic disease.

First, based on the low local recurrence rate of 5%, the use of TSS as primary treatment can be considered in case of juvenile tGrCT and in selected cases with small adult tGrCTs. However, the evidence is limited and only based on a minority of the cases treated with TSS. Patients should be informed about the low risk of local recurrence and the requirement for a completion orchiectomy in case of angiolymphatic invasion. If TSS is to be used, follow-up with testicular ultrasound should be considered.

Second, to identify adult tGrCT with metastatic potential, a larger tumor size, presence of angiolymphatic invasion and presence of gynecomastia represent predictive variables with good discriminatory accuracy. Given the high negative predictive value of those risk and late onset of recurrences, no general recommendation for regular follow-up with cross-sectional imaging of the abdomen and chest can be given. Instead, we recommend regular follow-up examination by a uro-oncologist for patients with risk factors on an annual basis for an extended postoperative period of up to 10–15 years.

On the other hand, given the low risk of recurrence of 3%, we recommend patients without risk factors or juvenile tGrCT to be followed up by their general practitioner. Imaging and/or referral to a uro-oncologist is recommended in case of clinical suspicion. Our data do not support the use of germ-cell tumor markers such as AFP, HCG, LDH during the follow-up of tGrCT patients.

Third, the non-existence of data regarding adjuvant therapy for localized disease questions the use of any adjuvant therapies. The fact that in 80% the primary metastatic tGrCT landing site was the retroperitoneum adjuvant RPLND might have the potential to cure some patients with micro-metastatic disease. However, as our data do not provide evidence that tGrCT have a discreet step-wise progression involving a specific primary landing site, adjuvant RPLND is not recommended.

Fourth, although the experience in men with metastatic disease is limited, response to surgical resection of metastases and/or chemotherapy with BEP was observed. We, therefore, suggest an aggressive curative approach with surgery alone or in combination with cisplatin-based chemotherapy in case of incomplete resection.

### Limitations

The published literature only consists of retrospective case reports and small case series. Moreover, reports of clinicopathological features were often inconsistent, making this analysis prone to bias. Our search strategy was designed and reviewed both by clinicians as well as librarians and was predefined in a peer-reviewed protocol. However, the possibility remains that not all potentially relevant studies were identified, which could be classified as an additional source of bias. Given the limited number of seven metastatic events, we refrained from running multivariable regression analyses and larger datasets are needed to develop prediction models involving several risk factors. The current analysis provides a unique overview of the published experience with juvenile and adult tGrCT. It may help physicians differentiate between tGrCT with a lower or higher risk for metastatic disease and select the most appropriate treatment modality for tGrCT patients.

Due to the absence of prospective trials, we recently opened the OrphAn Testis Histologies (OATH) to provide more conclusive recommendations regarding clinical course, management and follow-up of these rare entities. We encourage collaborators to contribute data of patients with rare testis cancer histologies (http://bit.ly/OATH-registry).

## Electronic supplementary material

Below is the link to the electronic supplementary material.

Supplementary material 1 (PDF 1507 kb)

## Abbreviations

tGrCT: Testicular granulosa cell tumors
SCST: Sex cord-stromal tumors
WHO: World Health Organization
TSS: Testis sparing surgery
NOS: Not otherwise specified
PRISMA: Preferred reporting items for systematic reviews and meta-analysis
IQR: Interquartile range
SD: Standard deviation
ROC: Receiver operating curve
AFP: Alpha fetoprotein
HCG: Human chorionic gonadotropin
LDH: Lactate dehydrogenase
RPLND: Retroperitoneal lymph node dissection
BEP: Bleomycin, etoposide and cisplatin
RPLN: Retroperitoneal lymph nodes
HPF: High-power field
DC: Doxorubicin–cisplatin
OATH: OrphAn Testis Histologies
