# Supplementary material for: Risk factors and treatment outcomes of 239 patients with testicular granulosa cell tumors: a systematic review of published case series data
Source: J Cancer Res Clin Oncol. 2020 Jul 27;146(11):2829–41. doi: 10.1007/s00432-020-03326-3 (PMC7519920; doi:10.1007/s00432-020-03326-3)
Supplement: Supplementary file 1 — Supplementary material 1 (PDF 1507 kb) [file 432_2020_3326_MOESM1_ESM.pdf]

Suchresultate zur Studie *Non-germ-cell cancer of the testis*

Suchprotokolle:

- Embase 02052018.pdf
- Medline 02052018.pdf
- Cochrane 02052018.pdf
- Scopus 02052018.pdf
- Web of Science 02052018.pdf

	Deduplication	
	Before	after
Medline (including Epub Ahead of Print, In-Process & Other Non-Indexed Citations)	1868	1865
Embase	2131	593
Cochrane	5	4
Scopus	2331	863
Web of Science	1528	366
Pool	7863	**3691**

Reference files: non germ cell cancer testis.enlx

Before:

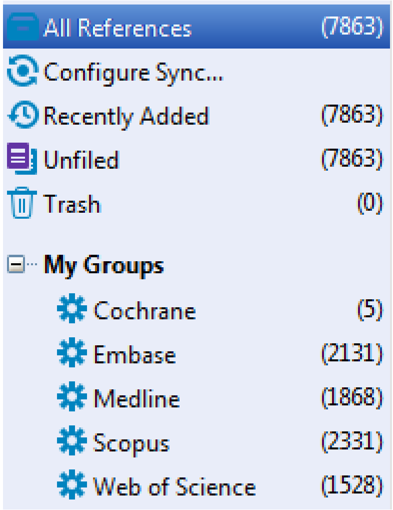

after:

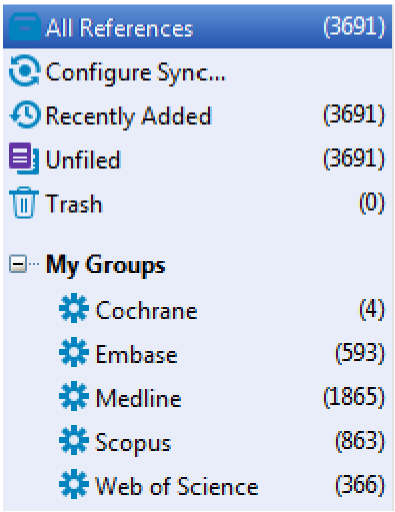

- **Search (/Cochranelibrary/search/quick)**
- **Search Manager (/Cochranelibrary/search/advanced)**
- **Medical Terms (MeSH) (/Cochranelibrary/search/mesh/quick)**
- **Browse (/Cochranelibrary/search/browse)**

To search an exact word(s) use quotation marks, e.g. "hospital" finds hospital; hospital (no quotation marks) finds hospital and hospitals; pay finds paid, pays, paying, payed)

Add to top

1.		#1	(sertoli or granulosa or "non-leydig*" or "sex cord" or stromal or "interstitial cell") near/3 (tumor* or tumour* or cancer* or carcinoma* or neoplasia* or malignan* or neoplasm*) or spermatocytic:ti,ab,kw (Word variations have been searched)	405
2.		#2	testis or testes or testicular or testicle*:ti,ab,kw (Word variations have been searched)	1493
3.	Edit	#3	#1 and #2 Enter terms for searc	5

Clear Strategy Search Help Highlight orphan lines

Save strategy

Strategy Name

Comments

Save Strategy

Embase®

Embase Session Results (2 May 2018)

No.	Query	Results
#4	#3 NOT ([animals]/lim NOT [humans]/lim) NOT [conference abstract]/lim	2131
#3	#1 AND #2	3032
#2	'testis'/exp OR testis:ti,ab OR testes:ti,ab OR testicular:ti,ab OR testicle*:ti,ab	169196
#1	'sertoli cell tumor'/exp OR 'granulosa cell tumor'/exp OR 'sex cord tumor'/exp OR 'spermatocytic seminoma'/exp OR (((sertoli OR granulosa OR 'non-leydig*' OR 'sex cord' OR stromal OR 'interstitial cell') NEAR/3 (tumor* OR tumour* OR cancer* OR carcinoma* OR neoplasia* OR malignan* OR neoplasm*)):ti,ab) OR spermatocytic:ti,ab	30632

ELSEVIER

© 2018 RELX Intellectual Properties SA. All rights reserved.
Embase, RELX Group and the RE symbol are trade marks of RELX Intellectual Properties SA, used under license.

Embase®

Ovid® Wolters Kluwer

My Account | Ask a Librarian | Support & Training | Help | Feedback | Logoff | University of Zürich

Search | Journals | Books | Multimedia | My Workspace | Mobile

Search History (4) View Saved

# ▲	Searches	Results	Type	Actions	Annotations
1	exp sex cord-gonadal stromal tumors/ or exp granulosa cell tumor/ or exp sertoli cell tumor/ or ((sertoli or granulosa or "non-leydig*" or "sex cord" or stromal or "interstitial cell") adj3 (tumor* or tumour* or cancer* or carcinoma* or neoplasia* or malignan* or neoplasm*)).ti,ab. or spermatocytic.ti,ab.	22701	Advanced	Display Results | More	
2	exp Testis/ or (testis or testes or testicular or testicle*).ti,ab.	131329	Advanced	Display Results | More	
3	1 and 2	2399	Advanced	Display Results | More	
4	3 not (animals not humans).sh.	1868	Advanced	Display Results | More	

Save | Remove | Combine with: AND | OR

Save All | Edit | Create RSS | View Saved

Basic Search | Find Citation | Search Tools | Search Fields | **Advanced Search** | Multi-Field Search

1 Resource selected | Hide | Change

Ovid MEDLINE(R) Epub Ahead of Print, In-Process & Other Non-Indexed Citations, Ovid MEDLINE(R) Daily and Ovid MEDLINE(R) 1946 to Present

Enter keyword or phrase (* or $ for truncation) — **Keyword** | Author | Title | Journal — Search

Limits *(close)* Include Multimedia | Map Term to Subject Heading

- Abstracts
- Full Text
- Core Clinical Journals (AIM)
- Structured Abstracts
- Review Articles
- Latest Update
- English Language
- Humans
- Pharmacologic Actions

Publication Year - | -

Additional Limits | Edit Limits

To search Open Access content on Ovid, go to Basic Search.

Options

Search Information

You searched:

3 not (animals not humans).sh.

Search terms used:

cancer*
carcinoma*
cell
cord
granulosa
granulosa cell tumor
interstitial
malignan*
neoplasia*
neoplasm*
non-leydig*
sertoli
sertoli cell tumor
sex
sex cord-gonadal stromal tumors
spermatocytic
stromal

Print | Email | Export | + My Projects | Keep Selected

All | Range | Clear | 10 Per Page | 1 | Go | Next ›

1. **Solid pseudopapillary neoplasm (SPN) of the testis: Comprehensive mutational analysis of 6 testicular and 8 pancreatic SPNs.**

Michalova K; Michal M; Sedivcova M; Kazakov DV; Bacchi C; Antic T; Miesbauerova M; Hes O; Michal M.

Annals of Diagnostic Pathology. 35:42-47, 2018 Apr 20.

[Journal Article]

UI: 29705715

Authors Full Name

Michalova, Kvetoslava; Michal, Michael; Sedivcova, Monika; Kazakov, Dmitry V; Bacchi, Carlos; Antic, Tatjana; Miesbauerova, Marketa; Hes, Ondrej; Michal, Michal.

Abstract | + My Projects | + Annotate

Abstract Reference | Complete Reference | Find Similar | Find Citing Articles | Full Text | S·F·X

testes
testicle*
testicular
testis
tumor*
tumour*

Search Returned:
1868 text results

Sort By:

-

Customize Display

Filter By

Add to Search History

Selected Only (0)

Years

All Years
Current year
Past 3 years
Past 5 years
Specific Year Range

Subject
Author
Journal
Publication Type

My Projects

+ New Project

No projects available.

2. **Prepubertal Malignant Large Cell Calcifying Sertoli Cell Tumor of the Testis.**

Li G; Lee MS; Kraft KH; Heider A.

Urology. 2018 Apr 04.

[Journal Article]

UI: 29626571

Authors Full Name
Li, Guanqun; Lee, Matthew S; Kraft, Kate H; Heider, Amer.

Abstract + My Projects + Annotate

Abstract Reference
Complete Reference
Find Similar
Find Citing Articles

3. **Spectrum of Findings in Orchiectomy Specimens of Persons Undergoing Gender Confirmation Surgery.**

Matoso A; Khandakar B; Yuan S; Wu T; Wang LJ; Lombardo KA; Mangray S; Mannan AASR; Yakirevich E.

Human Pathology. 2018 Mar 16.

[Journal Article]

UI: 29555572

Authors Full Name
Matoso, Andres; Khandakar, Binny; Yuan, Songyang; Wu, Tony; Wang, Li J; Lombardo, Kara A; Mangray, Shamlal; Mannan, Abul Ala Syed Rifat; Yakirevich, Evgeny.

Abstract + My Projects + Annotate

Abstract Reference
Complete Reference
Find Similar
Find Citing Articles

4. **The natural history of Leydig cell testicular tumours: an analysis of the National Cancer Registry.**

Nason GJ; Redmond EJ; Considine SW; Omer SI; Power D; Sweeney P.

Irish Journal of Medical Science. 2017 Jul 19.

[Journal Article]

UI: 28726031

Authors Full Name
Nason, G J; Redmond, E J; Considine, S W; Omer, S I; Power, D; Sweeney, P.

Abstract + My Projects + Annotate

Abstract Reference
Complete Reference
Find Similar
Find Citing Articles

5. **Testis-sparing surgery for benign testicular masses - diagnostics and therapeutic approaches.**

Paffenholz P; Held L; Loosen SH; Pfister D; Heidenreich A.

Journal of Urology. 2018 Mar 09.

[Journal Article]

UI: 29530784

Authors Full Name
Paffenholz, Pia; Held, Linn; Loosen, Sven H; Pfister, David; Heidenreich, Axel.

Abstract + My Projects + Annotate

Abstract Reference
Complete Reference
Find Similar
Find Citing Articles

6. **Spermatocytic Seminoma: A Report of 85 Cases Emphasizing its Morphologic Spectrum Including Some Aspects not Widely Known.**

Hu R; Ulbright TM; Young RH.

American Journal of Surgical Pathology. 2017 Dec 26.

[Journal Article]

UI: 29280854

Authors Full Name

Abstract Reference
Complete Reference
Find Similar
Find Citing Articles

Hu, Rong; Ulbright, Thomas M; Young, Robert H.

Abstract + My Projects + Annotate

7. **Microcystic Stromal Tumor of Testicle: First Case Report and Literature Review.**

Zhu P; Duan Y; Ao Q; Wang G.

Cancer Research & Treatment. 2017 Nov 24.

[Journal Article]

UI: 29169232

Authors Full Name
Zhu, Pengcheng; Duan, Yaqi; Ao, Qilin; Wang, Guoping.

Abstract + My Projects + Annotate

Abstract Reference
Complete Reference
Find Similar
Find Citing Articles

8. **Ovarian Sex Cord-stromal Tumors With Melanin Pigment: Report of a Previously Undescribed Phenomenon.**

Taylor J; McCluggage WG.

International Journal of Gynecological Pathology. 2017 Nov 14.

[Journal Article]

UI: 29140884

Authors Full Name
Taylor, Jennifer; McCluggage, W Glenn.

Abstract + My Projects + Annotate

Abstract Reference
Complete Reference
Find Similar
Find Citing Articles

9. **INSL3 Expression in Leydig Cell Hyperplasia and Leydig Cell Tumors.**

Lakis NS; Lombardo KA; Mangray S; Netto GJ; Salles D; Matoso A.

Applied Immunohistochemistry & Molecular Morphology. 2017 Oct 27.

[Journal Article]

UI: 29084059

Authors Full Name
Lakis, Nelli S; Lombardo, Kara A; Mangray, Shamlal; Netto, George J; Salles, Daniela; Matoso, Andres.

Abstract + My Projects + Annotate

Abstract Reference
Complete Reference
Find Similar
Find Citing Articles

10. **Ovarian Cancer Is an Imported Disease: Fact or Fiction?.**

Kuhn E; Kurman RJ; Shih IM.

Current Obstetrics & Gynecology Reports. 1(1):1-9, 2012 Mar.

[Journal Article]

UI: 22506137

Authors Full Name
Kuhn, Elisabetta; Kurman, Robert J; Shih, Ie-Ming.

Abstract + My Projects + Annotate

Abstract Reference
Complete Reference
Find Similar
Find Citing Articles
Full Text

All Range Clear 10 Per Page 1 Go Next ›

Print Email Export + My Projects Keep Selected

English Français Italiano Deutsch 日本語 繁體中文 Español 简体中文 한국어

© 2018 Ovid Technologies, Inc. All rights reserved. OvidSP_UI03.28.00.131, SourceID 110662

About Us Contact Us Terms of Use

Scopus

Document search

Compare sources >

Documents Authors Affiliations Advanced

Search tips ?

Search
(testis OR testes OR testicular OR testicle*)

Article title, Abstract, Keywords

E.g., "Cognitive architectures" AND robots

> Limit

Reset form Search

Search history

Combine queries... e.g. #1 AND NOT #3

3	(TITLE-ABS-KEY (((sertoli OR granulosa OR "non-leydig*" OR "sex cord" OR stromal OR "interstitial cell") W/3 (tumor* OR tumour* OR cancer* OR carcinoma* OR neoplasia* OR malignan* OR neoplasm*)) OR spermatocytic)) AND (TITLE-ABS-KEY ((testis OR testes OR testicular OR testicle*)))	2,331 document results
2	TITLE-ABS-KEY ((testis OR testes OR testicular OR testicle*))	173,367 document results
1	TITLE-ABS-KEY (((sertoli OR granulosa OR "non-leydig*" OR "sex cord" OR stromal OR "interstitial cell") W/3 (tumor* OR tumour* OR cancer* OR carcinoma* OR neoplasia* OR malignan* OR neoplasm*)) OR spermatocytic)	30,824 document results

^ Top of page

Brought to you by
The Scopus Team

SciVal offers quick, easy access to the research performance of 8,500 research institutions and 220 nations worldwide.
A ready-to-use solution with unparalleled power and flexibility, SciVal enables you to:

ELSEVIER
Terms and conditions Privacy policy

Copyright © 2018 Elsevier B.V. All rights reserved. Scopus® is a registered trademark of Elsevier B.V.

Cookies are set by this site. To decline them or learn more, visit our Cookies page.

RELX Group™

Web of Science

Search History

Web of Science Core Collection

Learn More

Save History / Create Alert | Open Saved History

Set	Results		Edit Sets	Combine Sets AND OR Combine	Delete Sets Select All Delete
# 3	1,528	#2 AND #1<br>*Indexes=SCI-EXPANDED, SSCI, A&HCI, CPCI-S, CPCI-SSH, BKCI-S, BKCI-SSH, ESCI, CCR-EXPANDED, IC*<br>*Timespan=All years*	Edit		
# 2	115,243	**TOPIC:** ((testis OR testes OR testicular OR testicle*))<br>*Indexes=SCI-EXPANDED, SSCI, A&HCI, CPCI-S, CPCI-SSH, BKCI-S, BKCI-SSH, ESCI, CCR-EXPANDED, IC*<br>*Timespan=All years*	Edit		
# 1	24,822	**TOPIC:** (((sertoli OR granulosa OR "non-leydig*" OR "sex cord" OR stromal OR "interstitial cell") NEAR/3 (tumor* OR tumour* OR cancer* OR carcinoma* OR neoplasia* OR malignan* OR neoplasm*)) OR spermatocytic)<br>*Indexes=SCI-EXPANDED, SSCI, A&HCI, CPCI-S, CPCI-SSH, BKCI-S, BKCI-SSH, ESCI, CCR-EXPANDED, IC*<br>*Timespan=All years*	Edit		

AND OR Combine | Select All Delete

© 2018 CLARIVATE ANALYTICS TERMS OF USE PRIVACY POLICY FEEDBACK
